# Complete mitochondrial genome of the Spanish toothcarp, *Aphanius iberus* (Valenciennes, 1846) (Actinopterygii, Aphaniidae) and its phylogenetic position within the Cyprinodontiformes order

**DOI:** 10.1007/s11033-022-08236-w

**Published:** 2023-01-17

**Authors:** Alfonso López-Solano, Tessa Lynn Nester, Silvia Perea, Ignacio Doadrio

**Affiliations:** 1 Department of Biodiversity and Evolutionary Biology, Museo Nacional de Ciencias Naturales (CSIC), Madrid, Spain; 2 Departamento de Zoología, Instituto de Biología, Universidad Nacional Autónoma de México, Tercer Circuito Exterior S/N, C.P. 04510 Ciudad de Mexico, Mexico

**Keywords:** Mitogenome, *Aphanius iberus*, Cyprinodontiformes, Phylogeny

## Abstract

**Background:**

The Spanish toothcarp (*Aphanius iberus* Valenciennes, 1846) is a small fish endemic to the eastern coastline of the Iberian Peninsula and is currently listed as “Endangered” (category IUCN: EN). It mainly inhabits brackish waters which can exhibit large fluctuations in temperature and salinity throughout the year. The genetics of *A. iberus* are not well-known since most studies have only evaluated the genetic structure of the species under a conservation framework in order to identify its potential conservation units. Different phylogenetic relationships of *Aphanius* have been published based on some particular genes. In the present study, the entire mitochondrial genome of *A. iberus* was obtained for the first time in the context of an *A. iberus* reference genome and a hypothesis regarding its phylogenetic position was considered.

**Methods and results:**

The mitogenome (a circular doble-stranded DNA sequence of 16,708 bp) was reconstructed and aligned against 83 Cyprinodontiformes and two outgroup taxa to identify the phylogenetic position of *A. iberus.* PartitionFinder was first used to test for the best evolutionary model and the phylogenetic analyses were performed using two methods: Maximun-Likelihood Approximation (IQ-Tree) and Bayesian inference (MrBayes). Our results show that *A. iberus* forms a sister group with *Orestias ascotanensis,* a cyprinodontiform species native to South America.

**Conclusions:**

The results were congruent with the traditional morphometric reconstructed trees and with a geological vicariant hypothesis involving Cyprinodontiformes where Aphaniidae is shown as a monophyletic family separated from the family Cyprinodontidae. The information gathered from this study is not only valuable for improving our understanding of the evolutionary history of *A. iberus*, but for future genomic studies involving the species.

## Introduction

The Spanish toothcarp (*Aphanius iberus* Valenciennes, 1846) is a small fish species endemic to the Mediterranean Coast of the Iberian Peninsula [1–3]. It belongs to the Cyprinodontiformes order [4] within the family Aphaniidae and although it can tolerate varying environmental conditions, including high temperatures and elevated salt concentrations [5], its populations have been in decline due to urbanism and agricultural exploitation which have dramatically transformed the eastern landscape of the Iberian Peninsula. Due to its fragmented distribution and its severe population decline in recent years as a result of habitat destruction, the Spanish toothcarp is currently listed as “Endangered” (IUCN: EN) [3, 6, 7], with about 20 isolated populations along the Iberian Mediterranean slope and it is currently restricted to salt marshes, coastal lagoons and natural springs referred to locally as Ullals [1, 3, 8]. To aid in the recovery of the species, conservation programs including two EU LIFE Projects (LIFE96 NAT/E/003118; LIFE04/NAT/ES/000035) are dedicated to the restoration of the natural habitat of *A. iberus* and the implementation of captive breeding programs. Currently, there is debate regarding the genus assignment of this species where some authors argue its belonging to the genus *Apricaphanius* Freyhof & YoĞurtÇuoĞlu [9], while others say it belongs to the genus *Aphanius* Nardo, 1827 [10]. Until further evidence arises, we have opted for the more conservative option in order to remain consistent with the International Code of Zoological Nomenclature, and therefore the genus, in this study, is referred to as *Aphanius*. We have also maintained *Aphanius farsicus* over *Esmaeilius persicus *which was recently proposed by Freyhof & YoĞurtÇuoĞlu [9].

Genetic studies involving *A. iberus* have mainly been focused on evaluating the genetic structure of the species under a conservation framework in order to identify its potential conservation units either with allozymes [8], partial gene sequences [11–14] or microsatellites [15]. However, the phylogenetic relationships within the order Cyprinodontiformes are still controversial and not yet clear especially since very few complete mitochondrial genomes are available causing some inconsistencies in relation to the phylogenetic position of *Aphanius* [10, 16, 17]. In this study, the entire mitochondrial genome of *A. iberus* was obtained for the first time in the context of an *A. iberus* reference genome sequence and a phylogenetic tree was constructed within the Cyprinodontiformes order. This new data will not only be valuable for improving our understanding of the evolutionary history of the species and its phylogenetic relationships, but also for future evolutionary and genomic studies.

## Methods

Three specimens of *A. iberus* were collected in El Palmar (Valencia, Spain) and euthanized following the appropriate protocols. The DNA was then isolated using the MagAttract HMW DNA isolation kit (Qiagen). The final elution was done in a volume of 100 μL. DNA was quantified using the Qubit High Sensitivity dsDNA Assay (Thermo Fisher Scientific).

Sequencing data within the context of a reference genome was obtained from PacBio and Illumina DNA sequencing in order to obtain the adequate assembly and annotation. Prior to PacBio library preparation, the sample was further purified and size-selected in order to maintain the largest fragments. Next, the recommended SMRTbell Express Template Prep Kit 2.0 (PacBio) was used to prepare the library, following the manufacturer’s instructions. The library was sequenced in a Sequel II sequencer (PacBio), using a SMRT Cell 8 M, under the Long-reads mode. A total of 7.3 million long reads (~ 9500) were obtained and quality-checked using the software SequelTools [18]. For posterior Illumina library preparation, the Illumina DNA Prep kit was used strictly following the manufacturer’s instructions. The fragment size distribution and concentration of the library was checked in the Agilent 2100 Bioanalyzer (using the Agilent HS DNA Kit). Then, the library was sequenced in a fraction of a NovaSeq PE150 flow cell, aiming for a total output of 50 gigabases, that yielded close to 435 million short paired-end reads (~ 150). The raw fastq files were quality-checked using the software FastQC v0.11.5 [19].

For the mitochondrial genome assembly NOVOPlasty v4.2 [20] was used as a seed-extend based assembler to reconstruct organelle genomes from whole-genome sequencing data, starting from a related or distant seed sequence. The Cytochrome C Oxidase Subunit 1 (COX1) gene of the species *Aphanius vladykovi* (NCBI Reference Sequence: MN702439.1) was selected as the seed, with a k-mer length of 33 bp.

The quality of the resulting mitochondrial assembly was then evaluated with the package QUAST 5.0.2 [21]. The number of contigs and the total length (in base pairs, bp) of the mitochondrial genome assembly are represented in Table 1. The mitogenome assembly generated was queried against the NCBI’s (National Center for Biotechnology Information) nr/nt (nucleotide) database using the Basic Local Alignment Search Tool BLAST. The best match found was the cyprinodontiform species *Cyprinodon variegatus* (Accession Number: KT288182.1) with a nucleotide identity percentage of 82% and the closely related *Orestias ascotanensis* exhibited a nucleotide identity percentage of 81.5%.

**Table 1:** Number of contigs and their total length

Assembly	Mitochondrial assembly
# contigs (≥ 0 bp)	1
# contigs (≥ 1000 bp)	1
# contigs (≥ 5000 bp)	1
# contigs (≥ 10,000 bp)	1
# contigs (≥ 25,000 bp)	0
# contigs (≥ 50,000 bp)	0
Total length (≥ 0 bp)	16,708
Total length (≥ 1000 bp)	16,708
Total length (≥ 5000 bp)	16,708
Total length (≥ 10,000 bp)	16,708
Total length (≥ 25,000 bp)	0
Total length (≥ 50,000 bp)	0
# contigs	1
Largest contig	16,708
Total length	16,708
GC (%)	45.06
N50	16,708
N75	16,708
L50	1
L75	1
'# N''s per 100 kbp'	0

The Mitochondrial genome annotation Server (MITOS2) [22] was used to automatically annotate the mitochondrial genome selecting RefSeq 63 Metazoa as the reference database and the vertebrate mitochondrial genetic code. The protein prediction method from Al Arab et al. [23] was enabled.

## Results

**Fig. 1:**
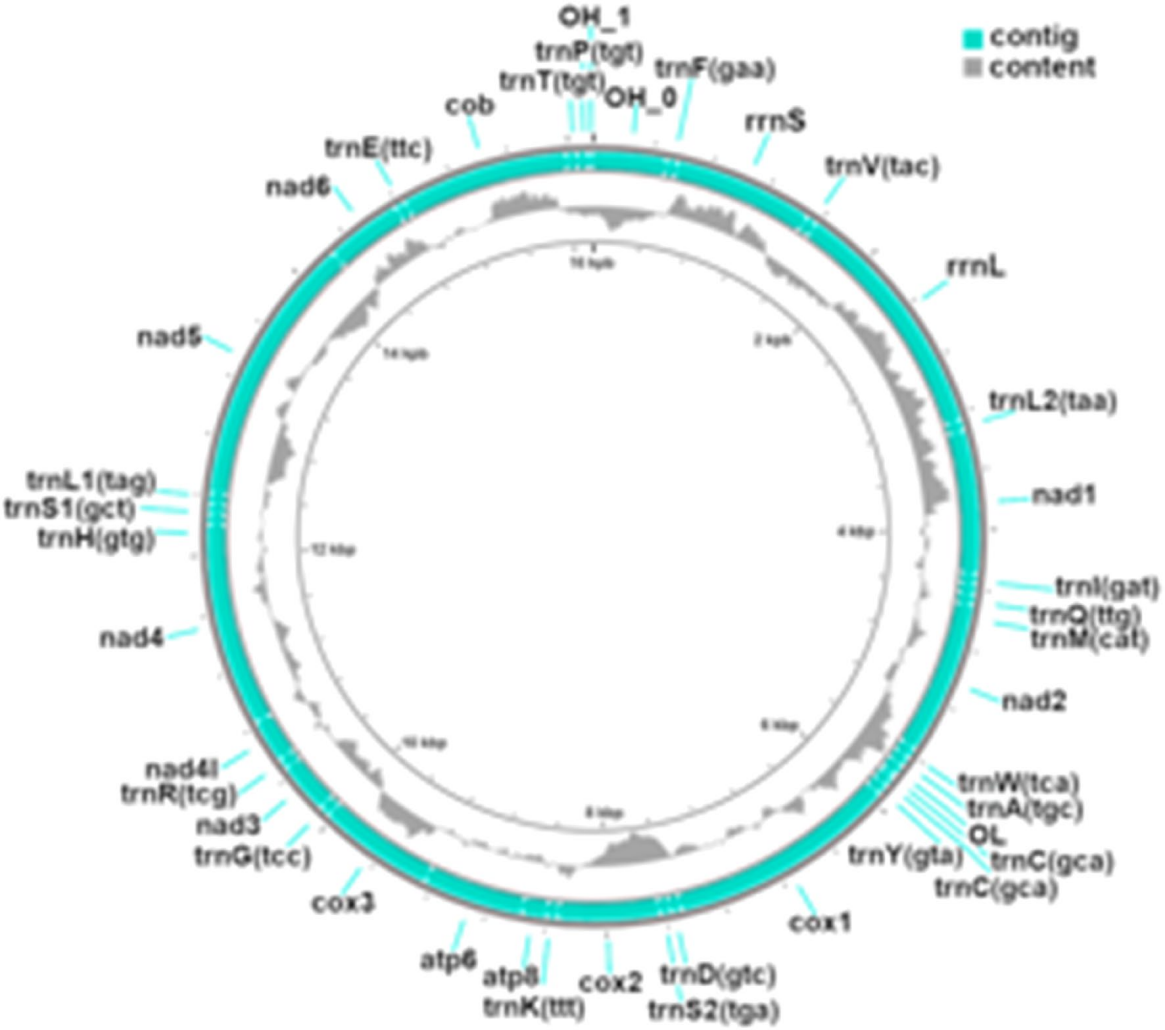
A graphical map of the mitochondrial genome of *A. iberus* showing the GC content and annotation results using the CGView server [24] (see Figure)

**Fig. 2:**
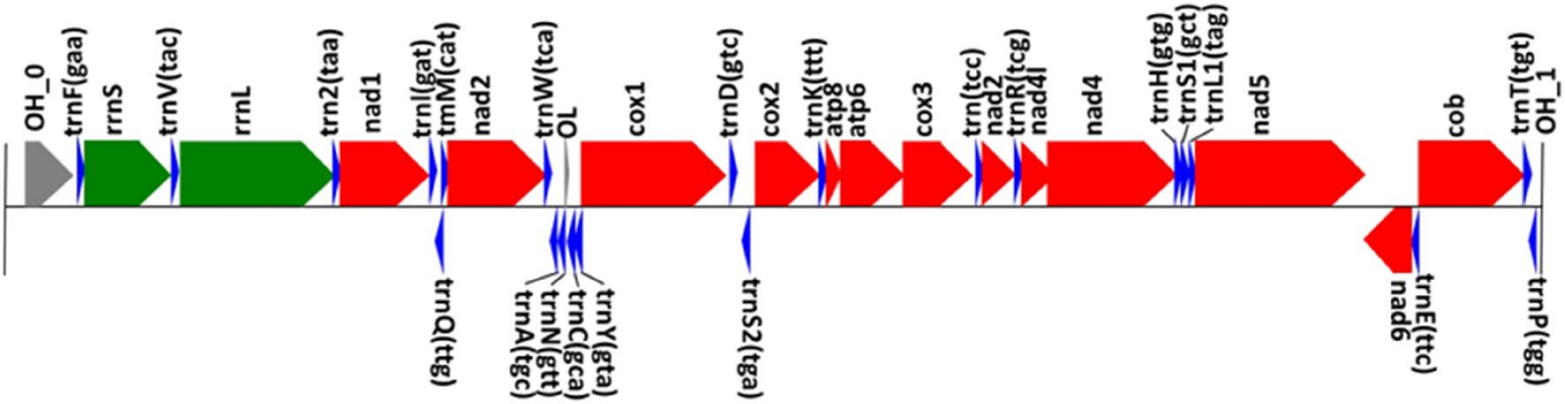
Linear representation of the *A. iberus* mitochondrial genome

The mitogenome of *Aphanius iberus* is a circular doble-stranded DNA sequence that is 16,708 bp long including 13 protein-coding genes, 22 transfer RNA genes, 2 ribosomal RNA genes, and the putative control region (Table 2, Figs. 1 and 2). The base percentage composition showed smaller G+C content (45.06%) compared to A+T content (54.94%). MITOS2 software annotated several peculiarities and found some OH overlaps in the following genes: (atp8,atp6):10; (nad4l,nad4):7; (nad5,nad6):4; (nad3,trnR):2; (trnI,trnQ):1; (trnQ,trnM):1; (nad2,trnW):1; (atp6,cox3):1; (cox3,trnG):1; (trnT,trnP):1.

**Table 2:** Organization of the *Aphanius iberus* mitogenome

Initial bp	Final bp	Type	Gene
223	730	D-loop	Control region
791	859	tRNA	tRNA-PHE
860	1807	rRNA	s-rRNA
1808	1879	tRNA	tRNA-VAL
1903	3565	rRNA	l-rRNA
3566	3639	tRNA	tRNA-LEU2
3640	4611	CDS	NADH dehydrogenase subunit 1
4613	4680	tRNA	tRNA-ILE
4750	4680	tRNA	tRNA-GLN
4750	4818	tRNA	tRNA-MET
4819	5865	CDS	NADH dehydrogenase subunit 2
5865	5935	tRNA	tRNA-TRP
6007	5939	tRNA	tRNA-ALA
6081	6009	tRNA	tRNA-ASN
6086	6119	rep_origin	Origin of L-strand replication
6184	6120	tRNA	tRNA-CYS
6255	6186	tRNA	tRNA-TYR
6257	7816	CDS	Cytochrome c oxidase subunit 1
7873	7943	tRNA	tRNA-ASP
8084	8014	tRNA	tRNA-SER2
8156	8846	CDS	Cytochrome c oxidase subunit 2
8847	8920	tRNA	tRNA-LYS
8922	9089	CDS	ATP synthase F0 subunit 8
9080	9763	CDS	ATP synthase F0 subunit 6
9763	10,548	CDS	Cytochrome c oxidase subunit 3
10,548	10,619	tRNA	tRNA-GLY
10,620	10,970	CDS	NADH dehydrogenase subunit 3
10,969	11,037	tRNA	tRNA-ARG
11,038	11,334	CDS	NADH dehydrogenase subunit 4L
11,328	12,708	CDS	NADH dehydrogenase subunit 4
12,709	12,777	tRNA	tRNA-HIS
12,778	12,845	tRNA	tRNA-SER1
12,856	12,928	tRNA	tRNA-LEU1
12,929	14,767	CDS	NADH dehydrogenase subunit 5
15,285	14,764	CDS	NADH dehydrogenase subunit 6
15,353	15,286	tRNA	tRNA-GLU
15,358	16,497	CDS	Cytochrome b
16,499	16,570	tRNA	tRNA-THR
16,637	16,570	tRNA	tRNA-PRO
16,678	16,708	D-loop	Control region

The mitochondrial genome was deposited on GenBank with the Accession Number OP884090; (BankIt2647138 Seq1). The Whole Genome Shotgun project has been deposited at DDBJ/ENA/GenBank under the accession JAPXFQ000000000. The version described in this paper is version JAPXFQ010000000. BioProject: PRJNA913687. BioSample: SAMN32303939.

**Fig. 3:**
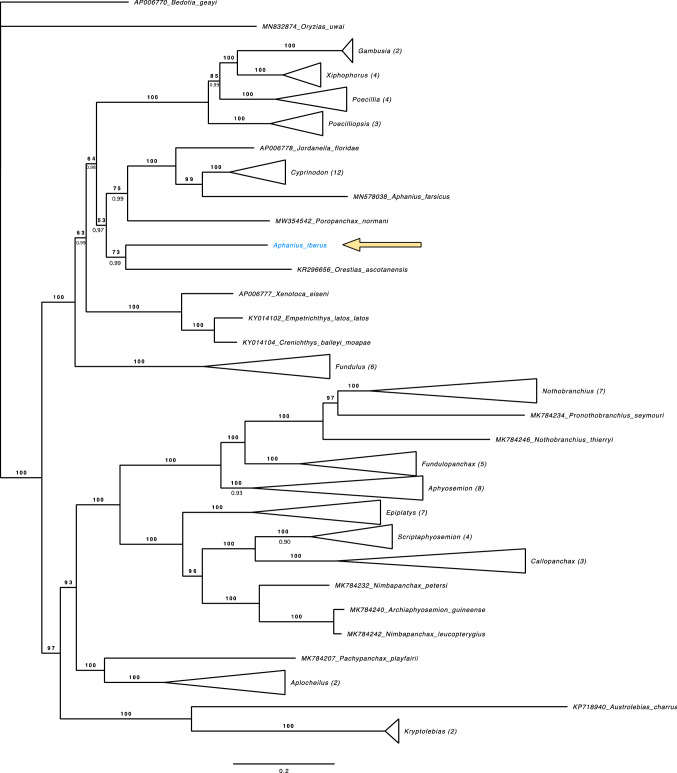
Phylogenetic tree rendered by Maximum likelihood and Bayesian inference based on the complete mitochondrial genome of 83 cyprinodontiform fishes. The phylogenetic position of *Aphanius iberus* is highlighted in red. The numbers on the branches indicate bootstrap (top of the branch) and posterior probability (bottom of the branch) values. (Color figure online)

A phylogenetic analysis was performed and included both coding regions (Nad1, Nad2, Cox1, Cox2, Atp8, Atp6, Cox3, Nad3, Nad4L, Nad4, Nad5, Nad6, CytB) and non-coding regions 12s (rrnS) and 16s (rrnL). Eighty-three cyprinodontiform species were selected and the mentioned mitogenome regions were downloaded from GenBank (Table 3) with the aim to evaluate the phylogenetic position of the mitogenome of *Aphanius iberus*. The following outgroup taxa were selected: *Oryzias uwai* (Accession Number: MN832874) and *Bedotia geayi* (Accession Number: AP006770) from the closely related Beloniformes and Atheriniformes orders respectively [25]. Geneious software [26] was used to align sequences using the MUSCLE alignment method including all of the 83 cyprinodontiform species, the two outgroup taxa, and the new mitogenome obtained for *Aphanius iberus* (Table 3). PartitionFinder2 software [27] was used to search for the best evolutionary model for each gene separately. The results revealed GTR+I+G as the best fit model for each gene, therefore, the entire mitogenome alignment was considered to belong to only one partition. Afterwards, two phylogenetic approximations were conducted based on two different methods. First, the Maximum-Likelihood reconstruction analysis was performed using the option MFP + MERGE in the IQ-TREE software [29]. The support for each node was evaluated with the SH-like approximate likelihood ratio test [30] and 1.000 ultrafast bootstrap (UFBoot2) approximations [31]. Then a Bayesian inference was performed with MrBayes [32]. Two analyses were run for 10,000,000 generations simultaneously, each with two parallel runs and four MCMC chains with a sampling frequency of 1000 generations. We rejected the first 25% of generations as burn-in and obtained the 50% majority rule consensus tree and the posterior probabilities (PP). The convergence of the runs was corroborated using Tracer v1.7.1 [33]. Finally, both phylogenetic trees inferred were imported into the software FigTree [34] and presented together with bootstrap values (over 100) and Bayesian probabilities (over 1) as the branch support (Fig. 3).

**Table 3:** Species selected from Genbank and classified by order and family with their respective accession numbers, authors and submission dates

Order, family	Species	Accession number	Author and submission date on GenBank
Cyprinodontiformes			
Rivulidae	*Austrolebias charrua*	KP718940	Gutierrez, V. et al. 28-JAN-2015
	*Kryptolebias marmoratus*	KT893707	Tatarenkov, A. et al. 08-OCT-2015
	*Kryptolebias hermaphroditus*	KX268503	Kim, H.-S. et al. 23-MAY-2016
Nothobranchiidae	*Nothobranchius furzeri*	EU650204	Reichwald, K. et al
	*Epiplatys dageti*	MK784208	Cui, R. et al. 11-APR-2019
	*Nothobranchius nubaensis*	MK784209	Cui, R. et al. 11-APR-2019
	*Aphyosemion kunzi*	MK784210	Cui, R. et al. 11-APR-2019
	*Scriptaphyosemion guignardi*	MK784211	Cui, R. et al. 11-APR-2019
	*Aphyosemion coeleste*	MK784212	Cui, R. et al. 11-APR-2019
	*Scriptaphyosemion bertholdi*	MK784213	Cui, R. et al. 11-APR-2019
	*Callopanchax toddi*	MK784214	Cui, R. et al. 11-APR-2019
	*Fundulopanchax amieti*	MK784215	Cui, R. et al. 11-APR-2019
	*Epiplatys togolensis*	MK784217	Cui, R. et al. 11-APR-2019
	*Epiplatys multifasciatus*	MK784219	Cui, R. et al. 11-APR-2019
	*Fundulopanchax scheeli*	MK784220	Cui, R. et al. 11-APR-2019
	*Scriptaphyosemion schmitti*	MK784221	Cui, R. et al. 11-APR-2019
	*Nothobranchius vosseleri*	MK784222	Cui, R. et al. 11-APR-2019
	*Epiplatys guineensis*	MK784223	Cui, R. et al. 11-APR-2019
	*Epiplatys lamottei*	MK784224	Cui, R. et al. 11-APR-2019
	*Aphyosemion cognatum*	MK784225	Cui, R. et al. 11-APR-2019
	*Fundulopanchax gardneri*	MK784226	Cui, R. et al. 11-APR-2019
	*Aphyosemion kouamense*	MK784227	Cui, R. et al. 11-APR-2019
	*Fundulopanchax sjostedti*	MK784228	Cui, R. et al. 11-APR-2019
	*Callopanchax monroviae*	MK784229	Cui, R. et al. 11-APR-2019
	*Nothobranchius kuhntae*	MK784230	Cui, R. et al. 11-APR-2019
	*Epiplatys spilargyreius*	MK784231	Cui, R. et al. 11-APR-2019
	*Nimbapanchax petersi*	MK784232	Cui, R. et al. 11-APR-2019
	*Nothobranchius ocellatus*	MK784233	Cui, R. et al. 11-APR-2019
	*Pronothobranchius seymouri*	MK784234	Cui, R. et al. 11-APR-2019
	*Fundulopanchax filamentosus*	MK784236	Cui, R. et al. 11-APR-2019
	*Aphyosemion australe*	MK784237	Cui, R. et al. 11-APR-2019
	*Aphyosemion gabunense*	MK784238	Cui, R. et al. 11-APR-2019
	*Callopanchax sidibeorum*	MK784239	Cui, R. et al. 11-APR-2019
	*Archiaphyosemion guineense*	MK784240	Cui, R. et al. 11-APR-2019
	*Aphyosemion cyanostictum*	MK784241	Cui, R. et al. 11-APR-2019
	*Nimbapanchax leucopterygius*	MK784242	Cui, R. et al. 11-APR-2019
	*Epiplatys grahami*	MK784243	Cui, R. et al. 11-APR-2019
	*Nothobranchius kafuensis*	MK784244	Cui, R. et al. 11-APR-2019
	*Scriptaphyosemion cauveti*	MK784245	Cui, R. et al. 11-APR-2019
	*Nothobranchius thierryi*	MK784246	Cui, R. et al. 11-APR-2019
	*Nothobranchius foerschi*	MK784247	Cui, R. et al. 11-APR-2019
	*Aphyosemion cameronense*	MK784248	Cui, R. et al. 11-APR-2019
Procatopodidae	*Poropanchax normani*	MW354542	Peng, Y. et al. 07-DEC-2020
Aplocheilidae	*Pachypanchax playfairii*	MK784207	Cui, R. et al. 11-APR-2019
	*Aplocheilus lineatus*	MK784216	Cui, R. et al. 11-APR-2019
	*Aplocheilus panchax*	NC_011176	Setiamarga, D. H. et al. 27-AUG-2008
Fundulidae	*Xenotoca eiseni*	AP006777	Setiamarga, D. H. et al. 06-APR-2004
	*Fundulus olivaceus*	AP006776	Setiamarga, D. H. et al. 06-APR-2004
	*Fundulus diaphanus*	FJ445394	Whitehead, A. 07-NOV-2008
	*Fundulus grandis*	FJ445396	Whitehead, A. 07-NOV-2008
	*Fundulus heteroclitus*	FJ445398	Whitehead, A. 07-NOV-2008
	*Fundulus notatus*	KP013106	Renshaw, M. A. et al. 21-OCT-2014
	*Fundulus zebrinus*	MW300328	Diver, T. A. et al. 27-NOV-2020
Goodeidae	*Empetrichthys latos latos*	KY014102	Jimenez, M. et al. 19-OCT-2016
	*Crenichthys baileyi moapae*	KY014104	Jimenez, M. et al. 19-OCT-2016
Poeciliinae	*Gambusia affinis*	AP004422	Miya, M. et al. 12-DEC-2001
	*Xiphophorus hellerii*	FJ226476	Bai, J.J. et al. 20-SEP-2008
	*Gambusia holbrooki*	KP013085	Renshaw, M.A. et al. 21-OCT-2014
	*Poeciliopsis occidentalis*	KP013108	Renshaw, M.A. et al. 21-OCT-2014
	*Poecilia reticulata*	KJ013505	Kong, X.F. et al. 31-DEC-2013
	*Poecilia formosa*	KT166983	Dang, X. et al. 17-JUN-2015
	*Poecilia latipinna*	KT175511	Stoeck, M. et al. 17-JUN-2015
	*Poecilia mexicana*	KT175512	Stoeck, M. et al. 17-JUN-2015
	*Xiphophorus couchianus*	KT594624	Zhang, K. et al. 25-AUG-2015
	*Poeciliopsis monacha*	KX229692	Jeon, Y. S. et al. 09-MAY-2016
	*Poeciliopsis sonoriensis*	MK860197	Mateos, M. et al. 27-APR-2019
	*Xiphophorus variatus*	MW934558	Eastis, A. N. et al. 15-APR-2021
	*Xiphophorus maculatus*	NC_011379	Setiamarga, D. H. et al. 17-OCT-2008
Cyprinodontidae	*Jordanella floridae*	AP006778	Setiamarga, D. H. et al. 06-APR-2004
	*Cyprinodon rubrofluviatilis*	EF442803	Crowl, T. M. et al. 29-MAR-2007
	*Cyprinodon tularosa*	KP013105	Renshaw, M. A. et al. 21-OCT-2014
	*Cyprinodon variegatus variegatus*	KR061357	Barcelon, B. R. et al. 04-APR-2015
	*Orestias ascotanensis*	KR296656	Quezada-Romegialli, C. et al. 28-APR-2015
	*Cyprinodon variegatus*	KT288182	Sheng, L. 14-JUL-2015
	*Cyprinodon nevadensis amargosae*	KU883631	Barcelon, B. R. et al. 08-MAR-2016
	*Cyprinodon diabolis*	KX061747	Lema, S. C. et al. 11-APR-2016
	*Cyprinodon julimes*	MG727890	Smith, N. L. et al. 24-DEC-2017
	*Cyprinodon elegans*	MW300326	Diver, T. A. et al. 27-NOV-2020
	*Cyprinodon macularius*	MW300330	Diver, T. A. et al. 27-NOV-2020
	*Cyprinodon bovinus*	MW300332	Diver, T. A. et al. 27-NOV-2020
	*Cyprinodon pecosensis*	MW300337	Diver, T. A. et al. 27-NOV-2020
	*Cyprinodon salinus salinus*	MW446237	Del Core, A. A. et al. 06-JAN-2021
Aphaniidae	*Aphanius persicus*	MN578038	Teimori, A. et al. 16-OCT-2019
	*Aphanius iberus*		This study
Beloniformes			
Adrianichthyidae	*Oryzias uwai* (outgroup)	MN832874	Ngamniyom, A. 14-DEC-2019
Atherinomorphae			
Atheriniformes	*Bedotia geayi* (outgroup)	AP006770	Miya, M. et al. 06-APR-2004

## Discussion

The phylogenetic analysis suggests the close relationship of *Aphanius iberus* with *Orestias ascotanesis.* The evolutionary relationship between *A. iberus* and *O. ascotanensis* exhibits high branch support with a bootstrap value of 73 and a posterior probability value of 0.99 (Fig. 3). This result was congruent with the traditional morphometric reconstructed trees [35–38] based on recent molecular studies [10, 17, 25] and with a geological vicariant hypothesis involving Cyprinodontiformes [16]. All of them revealed Aphaniidae (*Aphanius iberus*) as a monophyletic family separated from the Cyprinodontidae family (*Cyprinodon* and *Jordanella* genera, both included in our phylogenetic tree) [4] with a close proximity to Valenciidae [39]. Recently the complete mitogenome of *Aphanius* farsicus was published by Teimori & Motamedi, where they show *A. farsicus* positioned within the same evolutionary clade as the *Cyprinodon* and *Jordanella* genera [40]. Despite the genetic proximity of *A. farsicus* with *A. iberus* postulated by some studies [9, 10, 17], our analysis corroborated the close and highly supported mitochondrial relationship between *A. farsicus* and the *Cyprinodon*-*Jordanella* clade instead of with *A. iberus*. This latter issue questions the monophyly of the Aphaniidae family and poses the need for a further revision of this particular fish family within the order Cyprinodontiformes.

Mitochondrial DNA has proved to be a useful marker for deciphering the phylogenetic relationships at both inter- and intraspecific species levels [41]. On the one hand, its relatively fast evolutionary rate and nucleotide polymorphism has enough resolution to identify species and to even differentiate genetic groups within species. On the other hand, DNA recombination and the lack of complex genomic structures found in nuclear DNA (e.g. repeated elements, pseudogenes or introns) make for straightforward analyses. In fact, one of the mitochondrial genes, the cytochrome c oxidase subunit 1, has been designated as a barcode for species identification in many taxonomic studies [42, 43], but not without debate [44]. Some limitations of its use have been reported in the literature, including discrepancies with nuclear phylogenies [45, 46] or difficulty to identify hybridizations when these evolutionary events are present due to the maternal inheritance of the mitochondrial genome [47]. For these reasons, and due to the fact that only a few complete mitogenomes have ever been published for the genera *Aphanius* (two mitogenomes) and *Orestias* (one mitogenome), more research should be done in order to clarify the phylogenetic relationships of these genera and their species within the Cyprinodontiformes order in an evolutionary context.

## Conclusion

In this study, we have revealed that the genus *Aphanius s.l.* is not monophyletic. We have additionally proposed the close relationship between the western Mediterranean species *Aphanius iberus* and *Orestias ascotanensis*, a species from the Andean Region in South America. Our results are congruent with previous phylogenetic and biogeographical vicariant hypotheses involving Cyprinodontiformes. However, due to limitations of the mitochondrial genome and the fact that only a few were analyzed, further studies are required. Nevertheless, the information gained from this study is valuable for improving our understanding of the evolutionary history of *A. iberus* and for future genomic studies.

## References

[1] Oliva-Paterna FJ, Torralva M, Fernández-Delgado C (2006). Threatened fishes of the world: *Aphanius iberus* (Cuvier & Valenciennes, 1846) (Cyprinodontidae). Environ Biol Fish.

[2] Doadrio I (2001). Atlas y Libro Rojo de los Peces Continentales de España.

[3] Doadrio I, Perea S, Garzón-Heydt P, González L (2011). Ictiofauna continental española. Bases para su seguimiento.

[4] Freyhof J, Özuluǧ M, Saç G (2017). Neotype designation of *Aphanius iconii*, first reviser action to stabilise the usage of *A*. *fontinalis* and *A*. *meridionalis* and comments on the family group names of fishes placed in Cyprinodontidae (Teleostei: Cyprinodontiformes). Zootaxa.

[5] Lozano-Cabo F (1958). Contribución al conocimiento del “fartet” (*Aphanius iberus* C. y V.). Rev Acad Cien.

[6] Doadrio I, Carmona JA, Fernández-Delgado C (2002). Morphometric study of the Iberian *Aphanius* (Actinopterygii, Cyprinodontiformes), with description of a new species. Folia Zool.

[7] Crivelli AJ (2006) *Aphanius iberus*. The IUCN Red List of Threatened Species 2006: e.T1846A8299534. 10.2305/IUCN.UK.2006.RLTS.T1846A8299534.en

[8] Doadrio I, Perdices A, Machordom A (1996). Allozymic variation of the endangered killifish *Aphanius iberus* and its application to conservation. Environ Biol Fish.

[9] Freyhof J, YoĞurtÇuoĞlu B (2020). A proposal for a new generic structure of the killifish family Aphaniidae, with the description of *Aphaniops teimorii* (Teleostei: Cyprinodontiformes). Zootaxa.

[10] Esmaeili HR, Teimori A, Zarei F, Sayyadzadeh G (2020). DNA barcoding and species delimitation of the Old World tooth-carps, family Aphaniidae Hoedeman, 1949 (Teleostei: Cyprinodontiformes). PLoS ONE.

[11] Fernández-Pedrosa V, González A, Planelles M, Moya A, Latorre A (1995). Mitochondrial DNA variability in three Mediterranean populations of *Aphanius iberus*. Biol Conserv.

[12] Perdices A, Carmona JA, Fernández-Delgado C, Doadrio I (2001). Nuclear and mitochondrial data reveal high genetic divergence among Atlantic and Mediterranean populations of the Iberian killifish *Aphanius iberus* (Teleostei: Cyprinodontidae). Hered.

[13] Araguas RM, Roldán MI, García-Marín JL, Pla C (2007). Management of gene diversity in the endemic killifish *Aphanius iberus*: revising operational conservation units. Ecol Freshw Fish.

[14] Pappalardo AM, González EG, Tigano C, Doadrio I, Ferrito V (2015). Comparative pattern of genetic structure in two Mediterranean killifishes *Aphanius fasciatus* and *Aphanius iberus* inferred from both mitochondrial and nuclear data. J Fish Biol.

[15] González EG, Cunha C, Ghanavi HR, Oliva-Paterna FJ, Torralva M, Doadrio I (2018). Phylogeography and population genetic analyses in the Iberian toothcarp (*Aphanius iberus* Valenciennes, 1846) at different time scales. J Hered.

[16] Hrbek T, Meyer A (2003). Closing of the Tethys Sea and the phylogeny of Eurasian killifishes (Cyprinodontiformes: Cyprinodontidae). J Evol Biol.

[17] Pohl M, Milvertz FC, Meyer A, Vences M (2015). Multigene phylogeny of cyprinodontiform fishes suggests continental radiations and a rogue taxon position of *Pantanodon*. Vertebr Zool.

[18] Hufnagel DE, Hufford MB, Seetharam AS (2020). SequelTools: a suite of tools for working with PacBio Sequel raw sequence data. BMC Bioinform.

[19] Andrews S (2010) FastQC: a quality control tool for high throughput sequence data

[20] Dierckxsens N, Mardulyn P, Smits G (2017). NOVOPlasty: de novo assembly of organelle genomes from whole genome data. NA Res.

[21] Gurevich A, Saveliev V, Vyahhi N, Tesler G (2013). QUAST: quality assessment tool for genome assemblies. Bioinform.

[22] Bernt M, Donath A, Jühling F, Externbrink F, Florentz C, Fritzsch G, Püt J, Middendorf M, Stadler PF (2013). MITOS: improved de novo metazoan mitochondrial genome annotation. Mol Phylogen Evol.

[23] Al Arab M, Zu Siederdissen CH, Tout K, Sahyoun AH, Stadler PF, Bernt M (2017). Accurate annotation of protein-coding genes in mitochondrial genomes. Mol Phylogen Evol.

[24] Stothard P, Wishart DS (2004). Circular genome visualization and exploration using CGView. Bioinform.

[25] Betancur-R R, Wiley EO, Arratia G, Acero A, Bailly N (2017). Phylogenetic classification of bony fishes. BMC Evol Biol.

[26] Kearse M, Moir R, Wilson A, Stones-Havas S, Cheung M (2012). Geneious basic: an integrated and extendable desktop software platform for the organization and analysis of sequence data. Bioinform.

[27] Lanfear R, Frandsen PB, Wright AM, Senfeld T, Calcott B (2016). PartitionFinder 2: new methods for selecting partitioned models of evolution for molecular and morphological phylogenetic analyses. Mol Biol Evol.

[28] Kalyaanamoorthy S, Minh B, Wong T (2017). ModelFinder: fast model selection for accurate phylogenetic estimates. Nat Methods.

[29] Nguyen L-T, Schmidt HA, von Haeseler A, Minh BQ (2015). IQ-TREE: A fast and effective stochastic algorithm for estimating maximum likelihood phylogenies. Mol Biol Evol.

[30] Guindon S, Dufayard JF, Lefort V, Anisimova M, Hordijk W, Gascuel O (2010). New algorithms and methods to estimate maximum-likelihood phylogenies: assessing the performance of PhyML 3.0. Syst Biol.

[31] Hoang DT, Chernomor O, von Haeseler A, Minh BQ, Vinh LS (2018). UFBoot2: improving the ultrafast bootstrap approximation. Mol Biol Evol.

[32] Ronquist F, Teslenko M, Van Der Mark P, Ayres DL (2012). MrBayes 3.2: efficient Bayesian phylogenetic inference and model selection across a large model space. Syst Biol.

[33] Rambaut A, Drummond AJ, Xie D, Baele G, Suchard MA (2018). Posterior summarization in Bayesian phylogenetics using Tracer 17. Syst Biol.

[34] Rambaut A (2012). FigTree v1.4.

[35] Parenti LR (1980). A phylogenetic and biogeographic analysis of cyprinodontiform fishes (Teleostei, Atherinomorpha).

[36] Parenti LR (1984). A taxonomic revision of the killifish genus *Orestias* (Cyprinodontiformes, Cyprinodontidae). Ibid.

[37] Parker A (1991). Molecular evolutionary genetics of Cyprinodontiform fishes.

[38] Parker A, Kornfield I (1995). Molecular perspective on evolution and zoogeography of cyprinodontid killifishes (Teleostei; Atherinomorpha). Copeia.

[39] Quezada-Romegialli C, Guerrero CJ, Véliz D, Vila I (2015). The complete mitochondrial genome of the endemic and threatened killifish *Orestias ascotanensis*, Parenti, 1984 (Cyprinodontiformes, Cyprinodontidae) from the high andes. Mitochondrial DNA Part A.

[40] Teimori A, Motamedi M (2019). The first complete mitochondrial genome sequence in the genus *Aphanius* (Teleostei). J Ichthyol.

[41] Kowalczyk M, Staniszewski A, Kaminska K, Domaradzki P, Horecka B (2021). Advantages, possibilities, and limitations of mitochondrial DNA analysis in molecular identification. Folia Biol.

[42] Hebert PDN, Ratnasinghams S, Dewaard JR (2003). Barcoding animal life: cytochrome c oxidase subunit 1 divergences among closely related species. Proc Biol Sci.

[43] Dawnay N, Ogden R, McEwing R, Carvalho GR, Thorpe RS (2007). Validation of the barcoding gene COI for use in forensic genetic species identification. Forensic Sci Int.

[44] Dupuis JR, Roe AD, Sperling FAH (2012). Multi-locus species delimitation in closely related animals and fungi: one marker is not enough. Mol Ecol.

[45] Perea S, Sousa-Santos C, Robalo J, Doadrio I (2020). Multilocus phylogeny and systematics of Iberian endemic *Squalius* (Actinopterygii, Leuciscidae). Zoo Scripta.

[46] Poroshina AA, Sherbakov DY, Peretolchina TE (2020). Diagnosis of the mechanisms of different types of discordances between phylogenies inferred from nuclear and mitochondrial markers. Vavilovskii Zhurnal Genet Selektsii.

[47] Funk DJ, Omland KE (2003). Species-level parahyly and polyphyly: Frequencyy, causes, and consequences, with insights from animal mitochondrial DNA. Ann Rev Ecol Syst.

